# Hyaluronidase Availability Beyond the Aesthetic Office: A Shared Responsibility for Safety in Filler Complications

**DOI:** 10.1093/asj/sjaf018

**Published:** 2025-01-29

**Authors:** Daniel P Zaki, Joseph M Firriolo, Idean Roohani, Lee L Q Pu

We read with great interest the article titled The Use of Hyaluronidase in Aesthetic Practice: A Comparative Study of Practitioner Usage in Elective and Emergency Situations, published in the January 2024 issue of *Aesthetic Surgery Journal*.^1^ We commend the authors for their insightful exploration of hyaluronidase (HYAL) use in aesthetic practices and their emphasis on the critical nature of its availability.

We were particularly drawn to the discussion highlighting the variability in HYAL storage, preparation, and beliefs regarding adverse events due to the absence of standardized guidelines. As researchers who have conducted a comprehensive survey on the immediate availability of HYAL in emergency settings, we believe our findings could contribute to this ongoing discussion.

In our study, Hyaluronidase Availability in Emergency Rooms: A Statewide Analysis, published in *Facial Plastic Surgery & Aesthetic Medicine*, 25(2), we surveyed 330 emergency departments across California and achieved a response rate of 89.7%.^2^ Although it is best to stock an on-site reserve of HYAL as the authors of this article poignantly recognize, we also acknowledge that large quantities of on-site HYAL may not be feasible for all injectors, and as a result, local emergency rooms (ERs) form the final line of defense for intervention. Our results indicated that only 55% of ERs had immediate access to HYAL, a concerning statistic given the potential for patients to present to ERs with ischemic complications from hyaluronic acid (HA) fillers. Among the 66 adult trauma centers, 69.7% reported having HYAL on-site (Table 1). Although aesthetic patients presenting to ERs may be atypical, it remains a real possibility, underscoring the need for emergency preparedness—especially with the ongoing proliferation of various non–plastic surgeons and nonphysicians who inject HA (but don’t take calls) and can leave people with few other options than the ER.

**Table 1.** Hyaluronidase Availability in California Trauma Centers and Children's Hospitals

	Total ERs	Total response rate (%)	Total who did not carry HAse (%)	Total who carried HAse (%)	Carried 1 vial of HAse (%)	Carried 2-10 vials of HAse (%)	Carried 11 or more vials of HAse (%)
Adult trauma centers	73	66 (90.4)	20 (30.3)	46 (69.7)	2 (4.3)	30 (65.2)	10 (21.7)
Level I	14	12 (85.7)	3 (25.0)	9 (75.0)	1 (11.1)	4 (44.4)	3 (33.3)
Level II	35	32 (91.4)	8 (25.0)	24 (75.0)	0 (0.0)	19 (79.2)	3 (12.5)
Level III	13	13 (100.0)	2 (15.4)	11 (84.6)	1 (9.1)	6 (54.5)	3 (27.3)
Level IV	11	9 (81.8)	7 (77.8)	11 (22.2)	1 (0.0)	6 (50.0)	3 (50.0)
Pediatric trauma centers	16	15 (93.8)	3 (20.0)	12 (80.0)	1 (8.3)	6 (50.0)	4 (33.3)
Level I	8	7 (87.5)	1 (14.3)	6 (85.7)	0 (0.0)	2 (33.3)	2 (33.3)
Level II	8	8 (100.0)	2 (25.0)	6 (75.0)	1 (16.7)	4 (66.7)	2 (33.3)
Children's hospitals	25	18 (72.0)	4 (22.2)	14 (77.8)	1 (7.1)	8 (57.1)	5 (35.7)

Republished from Zaki et al, with permission from the publisher, Mary Ann Liebert, Inc.^2^ ERs, emergency rooms; HAse, hyaluronidase.

**Figure 1.**
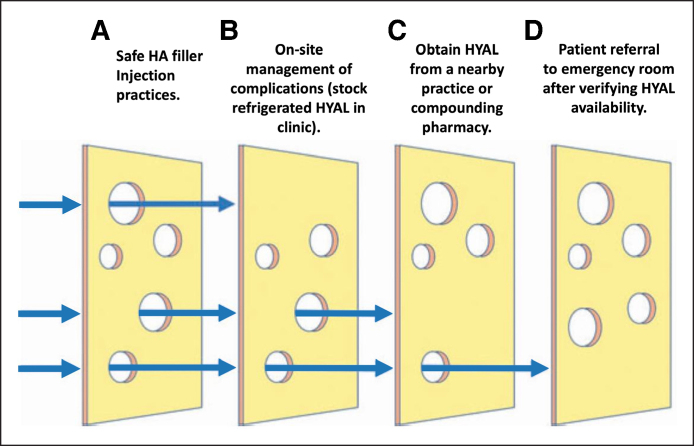
The Swiss Cheese Model for the prevention and management of ischemic complications secondary to hyaluronic acid filler injection. Republished from Zaki et al, with permission from the publisher, Mary Ann Liebert, Inc.^2^

We proposed a set of recommendations with a Swiss cheese model to enhance safety and preparedness (Figure 1):

1. Safe HA filler injection practices: Emphasizing proper techniques and protocols.
2. On-site management of complications: Advocating for the refrigeration and stocking of HYAL in clinics.
3. Collaboration with nearby practices or pharmacies: Ensuring access to HYAL through local networks.
4. Patient referral to emergency rooms: Verifying HYAL availability in ERs before referrals and considering children's hospitals, which are often better equipped with HYAL for treating extravasation injuries.

In our study, we sought to identify reliable predictors of HYAL's immediate availability and the critical role of ERs as the final safety net in managing severe complications. We encourage injectors to be methodical about patient referrals, confirm HYAL availability in ERs, and consider the use of extravasation kits.

Additionally, we noted the authors’ comment on the absence of evidence-based recommendations regarding HYAL concentration, dosing, and treatment intervals. Although we recognize the variability in protocols and need for consensus, there are several studies (both preclinical and clinical) that offer valuable insights into effective HYAL dosing protocols. For instance, ex vivo studies suggest that 1 to 2 vials of HYAL (150-300 IU) can manage localized ischemia, while higher doses (500-1500 IU) may be required depending on the extent of vascular occlusion and/or injury.^3^ Additionally, Schelke and colleagues demonstrated success with low doses of HYAL when a filler embolus was precisely targeted with the assistance of ultrasound technology.^4^ Other investigators have advocated for higher repeated multiplane injections at various depths to ensure resolution.^5^ DeLorenzi recommends HYAL dosing in the range of 500 to 1500 IU, depending on the pattern of ischemic injury observed.^6^ Repeated high-dose HYAL administration is much more likely to exhaust HYAL stores, which not only further emphasizes the need for prompt access to HYAL but also its availability in large quantities. Coupled with the authors’ commentary concerning the absence of clear guidelines with respect to HYAL storage once reconstituted, our findings further emphasize the necessity for ready access to HYAL in adequate quantities.

Again, we congratulate the authors on their valuable contribution to bridging gaps in this subject matter. We hope our findings and recommendations can further enrich the discourse on HYAL use in aesthetic practice and emergency preparedness, ultimately enhancing patient safety and outcomes. Thank you for considering our perspectives.
